# Loss of NRF2 During Aging Contributes to Myocardial Functional Decline

**DOI:** 10.3390/antiox15060672

**Published:** 2026-05-27

**Authors:** Lenee Shrestha, Yingying Lu, Wujing Dai, Suizi He, Daniel Wurm, Mingyi Wang, Judy Muller-Delp, Ling Ling An, Qin M. Chen

**Affiliations:** 1 Department of Pharmacy Practice and Science, College of Pharmacy, University of Arizona, 1295 N. Martin Ave, Tucson, AZ 85721, USA; lshrestha@arizona.edu (L.S.); wujingdai@outlook.com (W.D.); suizihe@arizona.edu (S.H.); wurm@arizona.edu (D.W.); 2 Graduate Interdisciplinary Program of Physiological Sciences, University of Arizona, Tucson, AZ 85721, USA; 3 Interdisciplinary Program in Statistics and Data Science, University of Arizona, Tucson, AZ 85721, USA; ylu1@arizona.edu (Y.L.);; 4 Laboratory of Cardiovascular Science, National Institute on Aging, Baltimore, MD 21224, USA; mingyiw@grc.nia.nih.gov; 5 Department of Physiology and Functional Genomics, University of Florida, 1600 SW Archer Road, Gainesville, FL 32603, USA; mullerdelp@vet.k-state.edu; 6 Department of Biosystems Engineering, University of Arizona, 1177 E. 4th Street, Tucson, AZ 85721, USA

**Keywords:** humans, monkeys, rats, mice, antioxidant and detoxification genes

## Abstract

Aging is a significant risk factor for cardiovascular diseases. The prevalence of heart failure increases with age, making it a leading cause of morbidity and mortality. We investigated age-associated changes in expression of Nuclear Factor (Erythroid-derived 2)-Like 2 (NFE2L2 or NRF2) in the myocardium of humans, rhesus monkeys, Fischer rats, and C57BL/6 mice. NRF2 is a transcription factor that orchestrates the expression of genes involved in antioxidant and detoxification responses. Analyses of RNA-seq data from the Genotype-Tissue Expression (GTEx) project, which contains left ventricular samples from 294 male donors, revealed a trend of age-associated declines in NRF2 transcripts and several of its downstream genes (SOD1, SOD2, CAT, GCLM, and AKR1B). Age-dependent decreases in NRF2 protein expression were observed in the myocardium of Rhesus monkeys and Fischer rats. To determine whether NRF2 loss contributes to myocardial aging, we evaluated cardiac function of NRF2 knockout mice (KO) at 19 and 24 months of age. At 19 months, the NRF2 KO mice exhibited diastolic dysfunction, characterized by an increased end-diastolic volume (EDV) and end-systolic volume (ESV), accompanied by a reduced ejection fraction (EF) and fractional shortening (FS), indicative of early onset of heart failure. The NRF2 KO mice displayed premature aging phenotypes and had reduced lifespans. Our findings support the trend of NRF2 signaling decline with age, and that loss of NRF2 accelerates the maladaptive cardiac remodeling and functional deterioration associated with aging.

## 1. Introduction

Aging is a complex biological process characterized by a gradual decline in physiological function across multiple organ systems. In the United States, the population aged 65 years and older is projected to increase by 47%, from 58 million in 2022 to 82 million by 2050 (U.S. Census Bureau). Among age-related disorders, cardiovascular disease (CVD) is one of the most prevalent conditions, often leading to heart failure [1]. Heart failure imposes a substantial healthcare burden and carries high morbidity and mortality rates. Understanding the intricate relationship between aging and the onset or progression of CVD is therefore critical for developing effective strategies to prevent and treat heart failure.

Age-associated alterations in ventricular structure and function have been well documented in humans and experimental models [2,3,4,5]. In humans, aging is accompanied by an increase in the left ventricular (LV) mass and wall thickness, along with a reduction in the LV chamber dimension and the development of diastolic dysfunction. Similar age-related cardiac remodeling has been reproduced in laboratory animals [6,7,8]. These structural and functional changes collectively contribute to ventricular hypertrophy and a decline in cardiac output, predisposing aging hearts to the onset and progression of heart failure.

Cardiac aging and CVD are both closely associated with oxidative stress [9,10]. During aerobic metabolism, Reactive Oxygen Species (ROS) are generated as byproducts, which are normally removed by endogenous antioxidant systems to maintain cellular redox homeostasis. A master regulator of these antioxidant systems is NRF2, a transcription factor that controls the expression of genes containing Antioxidant Response Elements (AREs) in their promoters. Classic examples of such genes include NAD(P)H quinone oxidoreductase 1 (NQO1), glutamate–cysteine ligase (catalytic subunit GCLC and regulatory subunit GCLM), glutathione peroxidases, and glutathione transferases [11]. NRF2 also regulates the expression of superoxide dismutases (SOD1, SOD2) and catalase (CAT) [11,12]. Beyond its role in antioxidant and detoxification responses, NRF2 influences the expression of genes involved in autophagy, cellular repair, and tissue remodeling [13,14,15,16]. These functions depict the critical role of NRF2 in modulating oxidative stress responses and suggest its potential contribution to the biological processes of aging.

The human NRF2 gene encodes a protein of 605 amino acids with seven distinct functional domains. The N-terminal Neh2 domain interacts with the cysteine-rich region of Kelch-like ECH-associated protein 1 (KEAP1), which recruits the CUL3-RBX1 ubiquitin E3 ligase complex to mediate NRF2 ubiquitination and subsequent proteasomal degradation [17]. Under oxidative stress, oxidation of KEAP1 cysteine residues disrupts this interaction, allowing NRF2 to escape the complex and translocate to the nuclei, where it activates transcription of target genes. The Neh2 domain of NRF2 is highly conserved between species, exhibiting 100, 96.23 and 94.34% sequence homology to humans in monkeys, rats and mice, respectively (Figure S1). The conservative nature of NRF2 protein sequence indicates that the oxidation state of KEAP1 similarly governs NRF2 transcriptional activity across mammalian species.

Little is known about whether aging impairs NRF2 gene expression in humans. Although small animals provide valuable experimental models for studying human diseases, the genetic and physiological differences between rodents and humans prohibit a linear extrapolation. This underscores the importance of cross-species comparisons to determine whether NRF2 signaling becomes dysregulated during aging. Over the past decade, national efforts to establish large-scale databases of human transcriptomic sequences have yielded valuable resources. The Genotype-Tissue Expression (GTEx) project provides RNA-seq datasets that enable assessment of NRF2 expression and its downstream genes across 54 human tissues [18,19,20]. This human data can be compared to findings from closely related species, such as Rhesus monkeys, as well as to those from small laboratory animals, such as rats and mice. Furthermore, the availability of transgenic and knockout mouse models facilitates functional studies aimed at delineating the cause-and-effect relationship between NRF2 signaling and the aging process.

## 2. Materials and Methods

### 2.1. Analysis of Human Gene Transcripts

**Figure 1:**
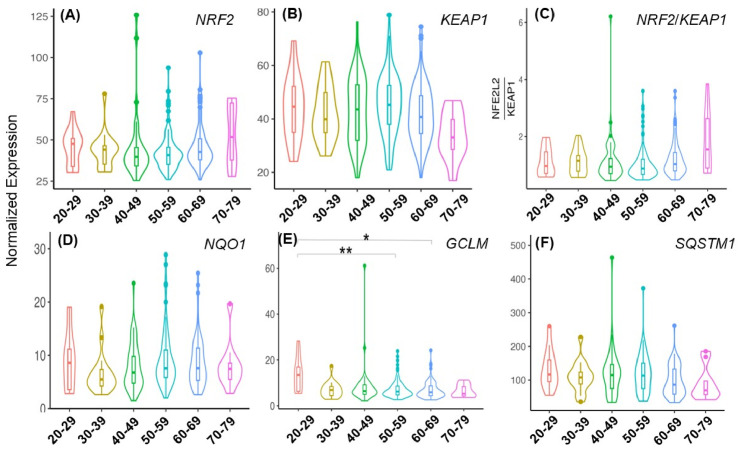
**NRF2, KEAP1, NQO1, GCLM, and SQSTM1 gene expression in human myocardial tissues among different age groups**. The level of each transcript was determined from RNA-Seq of the left ventricles of 294 male donors to the GTEx project. The violin plots show the distribution of normalized NRF2 (**A**), KEAP1 (**B**), NQO1 (**D**), GCLM (**E**), and SQSTM1 (**F**) transcript counts or the ratio of NRF2 transcripts over those of KEAP1 (**C**) from the donors across 6 age groups: 20–29 (n = 12), 30–39 (n = 17), 40–49 (n = 44), 50–59 (n = 107), 60–69 (n = 104) and 70–79 years old (n = 10). The data are presented as median values (center lines) with upper and lower quartiles (box limits). For the pairwise comparisons between age groups, the likelihood ratio tests were performed in edgeR. None of the changes meet the FDR-adjusted *p*-value < 0.05, except for GCLM. * or ** indicates FDR-adjusted *p*-value < 0.05 or <0.01, respectively.

RNA-Seq data were obtained from GTEx portal [20]. Full sample table from GTEx contains more than 54 tissues from 980 donors. Bam files corresponding to left ventricular tissues from 689 donors were extracted for read quality control (QC) analysis. Low-quality sequencing samples were excluded using following criteria: fewer than 10 million mapped reads, read mapping rate below 0.2, intergenic mapping rate greater than 0.3, base mismatch rate exceeding 0.01 for read mate 1 or 0.02 for read mate 2, and rRNA mapping rate greater than 0.3. After filtering, 431 donor samples met QC threshold and were retained for downstream analysis, of which 294 were male. Samples were grouped by age category, with number of samples per group indicated in Results and legend in Figure 1.

To determine the differential gene expression among age groups, we used edgeR (version 3.38.4), a widely adopted Bioconductor package for analyzing RNA-Seq read counts [21,22]. Lowly expressed genes were filtered out using the default algorithm in edgeR, retaining only those with more than 5 counts in at least 70% of the samples to ensure biological relevance. Normalization was performed using the trimmed mean of M values (TMM) method, and likelihood ratio tests were applied to compare each pair of age groups while controlling for the donor death category. The resulting *p*-values were adjusted using the Benjamini–Hochberg method to correct for multiple comparisons and control for false discovery rate (FDR) [23].

We compiled a list of over 1600 genes associated with the biological process of protein translation, based on information from published studies. This curated gene list was compared with the differentially expressed genes (DEGs) identified from the pairwise comparisons among age groups. The DEGs were selected using an adjusted *p*-value of less than 0.1 when comparing the 20–29 age group with the other age groups. To assess functional enrichment, we performed an over-representation analysis using the R package clusterProfile (version 4.4.4) [24]. This analysis evaluated whether particular biological pathways or gene functions were over-represented among the DEGs relative to expectations [24]. Such an enrichment analysis provides insight into the molecular processes that may be altered with aging, revealing the pathways potentially involved in age-associated biological changes.

### 2.2. Animal Tissues

All animals described in the study were cared for in accordance with NIH guidelines for the care and use of laboratory animals. The left ventricular free wall tissues of male rhesus monkeys ranging in age from 8.9 years to 25.5 years were obtained from the National Institute of Aging (NIA) nonhuman primate aging tissue repository. Samples were derived from the control group of the Age-associated Monkey Atherosclerosis study [25].

The protocol for animal use was reviewed and approved by the Institutional Animal Care and Use Committee (IACUC) of the University of Florida (for rats) and the University of Arizona (for mice). Male Fischer 344 rats were obtained from the NIA colony at 6 and 30 months of age, housed in a temperature-controlled environment, and provided standard rat chow and water ad libitum until the day of tissue harvest. Under isoflurane anesthesia (3–5% in O_2_ balanced), the heart was excised and snap frozen in liquid nitrogen for storage at −80 °C and shipment on dry ice. For extraction of protein and RNA, frozen heart samples were ground in a liquid nitrogen bath as described below.

The founder pairs of NRF2 KO mice were obtained from Dr. Donna Zhang’s laboratory with permission from Dr. Jefferson Y. Chan at the University of California, Irvine [26]. C57BL/6 wild-type (WT) and NRF2 knockout mice (KO) were bred in house in parallel from the same heterozygous ancestors. The mice were housed in a temperature-controlled room and provided standard chow and drinking water ad libitum. At 6, 19 and 24 months of age, the male animals were subjected to behavioral experiments and echocardiograms, with a one-week recovery period between the two procedures. To collect their tissues, the mice were injected intraperitoneally with heparin (300 U/kg) and euthanized by carbon dioxide (CO_2_) asphyxiation. Their hearts were excised and weighed to calculate the heart-to-body weight ratio.

### 2.3. RT-PCR to Measure Gene Transcripts

Total RNA was isolated from 50 mg (monkeys and rats) or 20 mg (mice) of snap-frozen left ventricular tissues following homogenization in Trizol Reagent. cDNA was synthesized from 1 µg of RNA using qScript cDNA SuperMIX kit (QuantaBio, Beverly, MA, USA). Quantitative PCR (qPCR) was performed using PowerUP SYBR Green Master Mix (A25777, Applied Biosystems, Waltham, MA, USA) on BioRad CFX96 Real-Time PCR system. Primer sets are shown in Table 1.

### 2.4. Western Blot for Protein Measurements

The snap-frozen left ventricular myocardial tissues (~50 mg) were ground into powder using a mortar and pestle placed in a liquid nitrogen bath. The powdered tissues were lysed by sonication in 1 mL of RIPA buffer (50 mM Tris pH 8.0, 150 mM NaCl, 0.5% sodium deoxycholate acid, 0.1% SDS, and 1% NP-40) with freshly added protease inhibitors containing AEBSF, Aprotinin, Bestatin, E-64, Leupeptin, and Pepstatin A in a cocktail (1:100 dilution, Millipore Sigma, Burlington, MA, USA). The protein concentration was measured using a Pierce Bicinchoninic Acid (BCA) Protein Assay Kit (ThermoFisher Scientific, Waltham, MA, USA). Equal amounts of protein (30 µg) were loaded onto 10% SDS-PAGE gels for separation and transferred to PVDF membranes using a Bio-Rad Trans-Blot Turbo Transfer System (Bio-Rad Laboratories, Hercules, CA, USA). The membranes were incubated with primary antibodies for NRF2 (16396-1-AP), KEAP1 (sc-15246), GCLM (sc-22755), NQO1 (sc-32793), SQSTM1 (sc-25575), GAPDH (sc-32233) or vinculin (VCL, sc-73614), followed by horseradish peroxidase (HRP)-conjugated secondary antibodies for chemiluminescent detection using a Bio-Rad ChemiDoc XRS+ imaging system. Densitometric analyses of the blots were performed using ImageJ (version 1.54g) software.

### 2.5. Echocardiography

C57BL/6 mice (male, at 6, 19, or 24 months of age) were anesthetized with 3% isoflurane and maintained with 1.5–2% isoflurane mixed in oxygen (USP, Phoenix, AZ, USA) during procedure. Mice were positioned in dorsal recumbence on heated platform to maintain body temperature at 35–37 °C. Transthoracic echocardiographic images were obtained using Vevo 3100 High-Resolution Imaging System (Visual-Sonics, Toronto, Canada) equipped with transducer (model MX550D) operating at frequency of 25–55 MHz, optimized for cardiac imaging of adult mice. Images were acquired at depth setting of 10–15 mm and stored as digital cine loops for offline analysis. Standard imaging planes, M-mode, Doppler recordings, and functional measurements were obtained in accordance with American Society of Echocardiography guidelines. Parasternal short-axis view of left ventricle (LV) was used to assess fractional shortening, ejection fraction, and ventricular dimensions or volumes. Left atrial dimensions were measured from long-axis view just below aortic valve leaflets. Peak velocity of passive LV filling (E, cm/s) and peak flow velocity during left atrial contraction (A, cm/s) were derived from mitral valve Doppler flow images obtained from tilted parasternal long-axis views. Sweep speed of 100 mm/s was applied for both M-mode and Doppler studies. Given positive correlation between heart rate and systolic performance, heart rates were maintained within target ranges during imaging: 425–500 beats/min for M-mode, 400–450 beats/min for B-mode, and 325–400 beats/min for Doppler studies. These ranges were achieved by adjusting concentration of isoflurane.

### 2.6. Histology

Hearts were perfused with 1 × PBS followed by 10% neutral buffered formalin for 5–10 min and fixed in 10% formalin for 24 h. After fixation, tissues were transferred to 70% ethanol solution and processed for hematoxylin and eosin (H&E) staining by iHisto Histopathology Support Laboratory (Salem, MA, USA). Paraffin-embedded hearts were sectioned transversely across ventricles and stained with H&E. Stained slides were examined under 2× magnification lens using Cell Image Multimode Reader Cytation 7 (BioTek Instruments, Inc., Winooski, VT, USA) or under 60× magnification lens using Olympus BX53 microscope (Olympus Corporation, Tokyo, Japan) equipped with DP72 digital camera. Images were acquired and analyzed using Gen5 (BioTek, 3.0) or Olympus cellSens (3.0, Olympus Corporation, Tokyo, Japan) software, respectively.

### 2.7. Open Field Training and Nesting

Male C57BL/6 mice at 6, 19, or 24 months of age were placed in 12 × 12-inch testing chambers and allowed to move freely for the duration of the test. Each animal was video monitored for 10 min, after which it was returned to their home cage. The video recordings were analyzed to determine the proportion of time spent on the perimeter versus the center of chamber, the total distance traveled, and the average velocity throughout the test session. All testing chambers were cleaned between animals to eliminate olfactory cues.

Approximately one hour before testing, the mice were transferred to individual cages with clean bedding and no environmental enrichment items to allow for habituation to the new environment. A pre-weighed nestlet was placed in the center of each cage, and nest-building behavior was evaluated after 12 h according to established guidelines for assessing nest construction in mice [27]. Any untorn nestlet material was reweighed to quantify the nest-building activity.

## 3. Results

### 3.1. NRF2, KEAP1, and Antioxidant or Detoxification Gene Expression in Human Aging

To address whether aging affects the expression of NRF2 and its downstream antioxidant or detoxification genes in humans, we divided a total of 294 male donors into six age groups—20–29 (n = 12), 30–39 (n = 17), 40–49 (n = 44), 50–60 (n = 107), 60–69 (n = 104) and 70–79 (n = 10)—for pairwise comparisons. Female samples were excluded due to the limited numbers in certain age groups and sex-dependent transcriptional differences.

For the NRF2 transcripts, the median expression levels exhibited a downward trend, although not statistically significant, from the 20–29 age group to the 40–49 age group, but appeared to rise from the 50–59 to the 70–79 age group (Figure 1A). Since NRF2 activity is negatively regulated by binding to KEAP1, we determined the KEAP1 transcript levels among the age groups. A non-significant decreasing trend was observed in the 70–79 age group (Figure 1B). This apparent decline in KEAP1 expression corresponded to a modest but non-statistically significant increase in the NRF2/KEAP1 transcript ratio in the 70–79 age group (Figure 1C).

NRF2 is known to regulate a panel of antioxidant enzymes and redox cycling proteins (NQO1, HMOX1, GPX3, GPX4, GSR, PRDX1, TXN, TXNRD1, and SRXN1) because these genes contain AREs within their promoters [28]. The transcriptional status of NRF2 also influences the expression of SOD1, SOD2, and CAT [11,13]. The transcripts of these genes were compared between age groups. Although downward trends with advancing age were observed for SOD1, SOD2, PRDX1, and SRXN1, these changes did not reach statistical significance, except for SOD2 in the 50–59 age group and SRXN1 in the 60–69 age group (*p* < 0.05, Figure S2). NQO1, a canonical marker of NRF2 activation, did not exhibit a consistent age-dependent change (Figure 1D).

The transcripts of NRF2 downstream detoxification genes were analyzed (Figure S3). Among these genes, GCLM showed significant decreases in the 50–59 and 60–69 age groups compared with the 20–29 age group (Figure 1E). Metal-binding proteins represent another class of NRF2 target genes. Ferritin consists of a heavy chain (FTH1) and a light chain (FTL). Neither FTH1 nor FTL showed significant age-dependent changes in expression (Figure S4). There are 10 metallothionein (MT) genes in humans, and their overall expression did not exhibit consistent age-related variation (Figure S4). However, several isoforms, MT1A, MT1M, MT1X, and MT2A, displayed significantly higher expression levels in the 70–79 age group (Figure S4).

NRF2 also regulates a subset of autophagy-associated genes, including SQSTM1 (p62), CALCOCO2, ULK1, ATG5, and GABARAPL1 [14,15]. Because LC3 plays a central role in autophagy, we included its gene, MAP1LC3B, in the analyses (Figure S5). Among these genes, SQSTM1 (p62) exhibited a non-significant but progressive decline with advancing age (Figure 1F). A significant decrease in MAP1LC3B was discovered in the 50–59 and 60–69 age groups (Figure S5).

Population-based proteomics datasets for human myocardial tissues are currently unavailable, thereby preventing direct assessment of NRF2 or its downstream effectors at the protein level. However, transcriptomic analyses of age-associated changes revealed that Gene Ontology (GO) analyses of biological processes identified “regulation of translation” as the top enriched GO term (Figure S6). Most genes classified under this category exhibited decreased transcript abundance (Figure S6B–E), suggesting an age-related decline in global protein translation. This supports the notion of reduced NRF2 protein synthesis with advancing age.

### 3.2. Aging-Associated Changes in NRF2 and Related Genes in Monkey and Rat Myocardial Tissues

**Figure 2:**
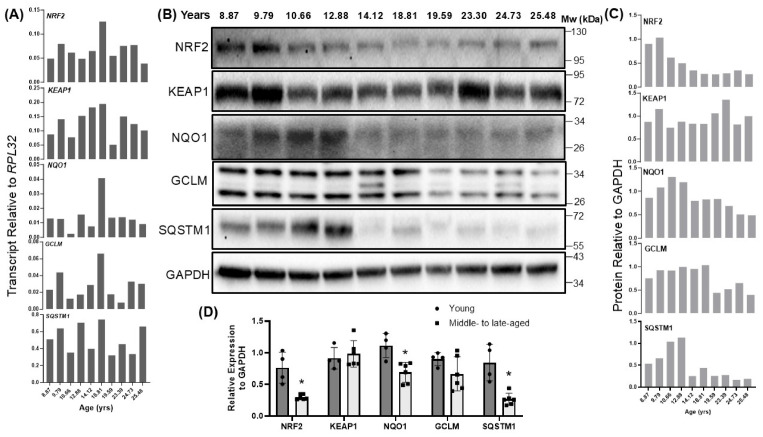
**Expression of NRF2 and its target genes in rhesus monkey myocardial tissue**. Average mRNA levels normalized to RPL32 of NRF2, KEAP1, NQO1, GCLM, and SQSTM1 obtained via RT-qPCR using total RNA extracted from myocardial tissues of male Rhesus monkeys, with ages ranging from 8.87 to 25.48 years old (**A**). Corresponding protein levels measured by Western blot analysis (**B**), with densitometric quantification by ImageJ (version 1.54g) using GAPDH as loading control (**C**). Each sample is from one monkey with its age indicated. Samples are divided into two age categories, young (8.87–12.88 years old) and middle- to late-aged (14.12–25.48 years old), for statistical comparisons (**D**). * indicates *p* < 0.05 by Student’s *t* test or Mann–Whitney non-parametric test.

Left ventricular tissues from male rhesus macaques were obtained through the NIA aging tissue repository, with ages ranging from 8.87 to 25.4 years old. The transcripts of NRF2, KEAP1, and representative NRF2 downstream genes (NQO1, GCLM, and SQSTM1) were analyzed by RT-qPCR. Transcript levels of NRF2, KEAP1, NQO1, GCLM, and SQSTM1 remained stable across the age samples (Figure 2A). When the NRF2 protein levels were measured, an age-associated decrease in the NRF2 protein level was observed, starting at 10.66 years old (Figure 2B,C). When the samples were grouped into young (8.87 to 12.8 years old) versus middle-to-late age categories (14.1 to 25.48 years old), the NRF2 protein showed a statistically significant decline in the middle-to-late age group (Figure 2D). The protein levels of NQO1, GCLM, and SQSTM1 similarly declined with age (Figure 2B–D). In contrast, the KEAP1 protein levels were largely unchanged (Figure 2B–D). These findings indicate that while the transcripts of NRF2 and related genes remain stable in monkey ventricular tissues, an age-related decline in NRF2 protein expression was detected.

**Figure 3:**
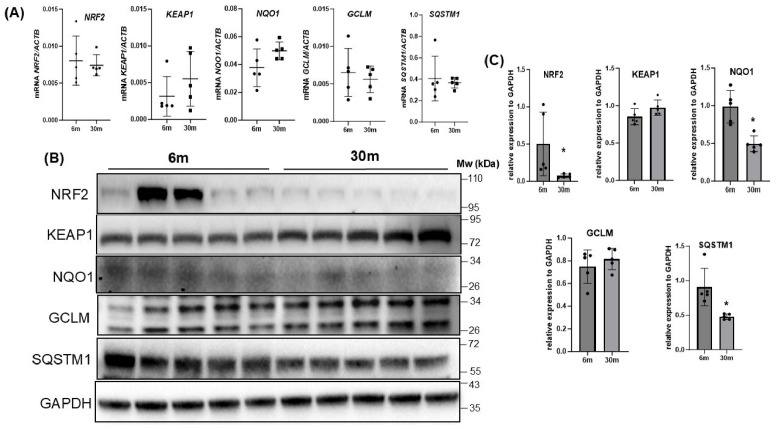
**Comparison of NRF2 mRNA or protein expression in young vs. old rat myocardial tissue**. Total RNA was collected from aged (30 months old, n = 5) and young (6 months old, n = 5) male Fischer 344 rats for RT-qPCR to measure transcripts of NRF2, KEAP1, NQO1, GCLM, and SQSTM1 (**A**). Western blots of corresponding genes were performed using the same set of tissue lysates (**B**). Intensities of bands from Western blots are quantified using ImageJ and normalized to GAPDH (**C**). Student’s *t*-test and Mann–Whitney test (for NRF2) are used for statistical analyses comparing 6-month-olds vs. 30-month-olds, with * indicating *p* < 0.05.

Myocardial tissues were collected from whole hearts of young (6 months old) and aged (30 months old) male Fischer 344 rats. The RT-qPCR analysis revealed no significant differences in the NRF2 transcript levels between the young and aged rats (Figure 3A). Similarly, transcripts of KEAP1, NQO1, GCLM, and SQSTM1 showed no significant age-related changes (Figure 3A). When protein levels of these genes were measured, the KEAP1 and GCLM protein levels remained unchanged, but NRF2, NQO1, and SQSTM1 showed significant decreases in the aged group (Figure 3B,C).

### 3.3. NRF2 Deficiency Accelerates Myocardial Aging

**Figure 4:**
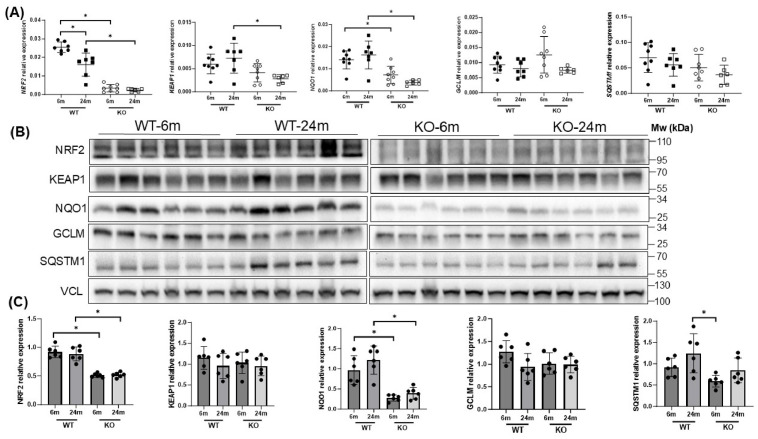
**Expression of NRF2 and its target genes in the myocardial tissues from young and aged wild-type and NRF2 KO mice**. NRF2, KEAP1, NQO1, GCLM, and SQSTM1 transcripts are quantified by RT-qPCR with normalization to GAPDH using total RNA extracted from myocardial tissues of 6-month-old and 24-month-old male wild-type and NRF2 KO mice (**A**). Western blot analyses measure proteins levels of the corresponding genes (**B**). Band intensities are quantified by ImageJ (version 1.54g) and normalized to vinculin (VCL) in 6-month-old and 24-month-old tissue samples (**C**). Bar graphs represent means ± SD, with * indicating *p* < 0.05 by one-way ANOVA test.

C57BL/6 mice are among the most commonly used experimental animal strains. When the myocardial tissues from 24-month-old male C57BL/6 mice were compared with those from 6-month-old mice, an approximate 30% reduction in NRF2 mRNA was observed (Figure 4A). However, measurements of NRF2 protein did not show a corresponding decrease (Figure 4B,C). KEAP1, NQO1, GCLM, and SQSTM1 did not exhibit significant age-associated changes at either the mRNA or protein levels in the wild-type mice (Figure 4).

NRF2 gene knockout (KO) mice have been established. These KO mice have normal physiological characteristics, enabling evaluation of whether NRF2 deficiency contributes to myocardial aging. These KO mice were generated by replacing part of exon 4 and all of exon 5 of the NRF2 gene encoding the C-terminal transactivation domains with the bacterial LacZ gene [26]. Loss of NRF2 gene expression was confirmed by undetectable NRF2 mRNA (Figure 4A). As expected, the NRF2 KO mice displayed a marked reduction in NQO1 mRNA expression (Figure 4A). Protein expression of NRF2 downstream targets was mostly consistent with their transcript levels (Figure 4B,C).

**Figure 5:**
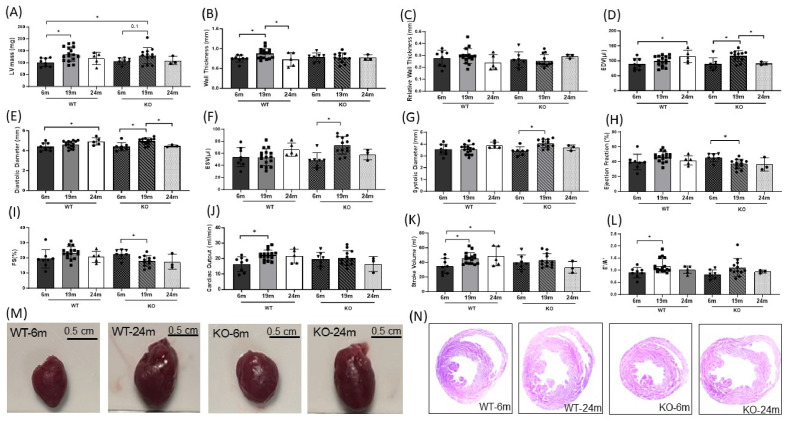
**Age-associated cardiac remodeling in wild-type and NRF2 KO mice**. Echocardiography was performed on C57BL/6 mice under anesthesia at 6, 19, or 24 months old. Standard imaging planes, M-mode, and Doppler analyses are used to evaluate cardiac function and structural parameters. Data are reported as means ± SD, with * indicating *p* < 0.05 by one-way ANOVA test (**A**–**L**). Representative images of excised hearts illustrate anatomical differences in aged animals (**M**). Transverse sections of heart ventricles are stained with hematoxylin and eosin (H&E) and observed using Cytation 7 Cell Image Multimode Reader under 2× magnification lens (**N**). EDV: end-diastolic volume; ESV: end-systolic volume; FS; fraction shortening; E’/A’: early (E)-to-late (A) transmitral flow velocity.

Cardiac function was assessed by echocardiography to evaluate the aging-associated structural and functional alterations. The wild-type mice exhibited cardiac remodeling characterized by an increased left ventricular (LV) mass and wall thickness, indicative of concentric hypertrophy at 19 months of age (Figure 5A,B). This remodeling was accompanied by a significant increase in cardiac output (Figure 5J). As the mice aged from 19 to 24 months, both the ventricular mass and wall thickness returned (Figure 5A,B). Meanwhile, the end-diastolic volume (EDV) and stroke volume increased at 24 months of age, suggesting ventricular dilation and progression towards eccentric hypertrophy (Figure 5D). Nevertheless, the cardiac output or ejection fraction did not show significant difference from those at 6 months old, indicating preserved contractile function.

Compared to the wild-type mice, the NRF2 KO mice exhibited eccentric hypertrophy at 19 months of age, evidenced by increases in the EDV, end-diastolic diameter, end-systolic volume (ESV) and end-systolic diameter (Figure 5D–G). At this age, the NRF2 KO mice showed significant reductions in their ejection fraction and fractional shortening (FS), indicating the onset of heart failure (Figure 5H,I). Overall, these echocardiographic findings demonstrate accelerated cardiac remodeling, ventricular dilation, and an age-associated decline in cardiac function, as shown in the 19-month-old NRF2 KO mice.

The excised hearts showed an enlarged cardiac size in both the wild-type and NRF2 KO mice at 24 months of age (Figure 5M). The older NRF2 KO mice exhibited a reduced body weight, which contributed to the elevated heart weight-to-body weight ratio (Figure S7A,B). The histological analysis confirmed an enlargement of the left ventricles (Figure 5N). Measurements of heart failure biomarkers BNP, ANP and β-MHC in the myocardial tissues support that the NRF2 KO mice were progressing towards heart failure at 24 months old (Figure S7C–E).

### 3.4. Accelerated Aging of NRF2 KO Mice

**Figure 6:**
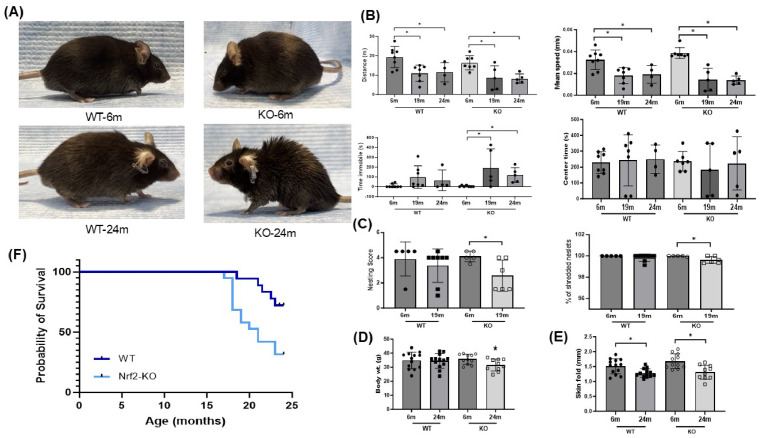
**Aged NRF2 KO mice exhibit an accelerated aging phenotype, impaired locomotor activity, and reduced lifespan**. Representative images of young (6 months old) and aged (24 months old) male wild-type and NRF2 KO mice were acquired to demonstrate overall appearance (**A**). An open field Test was performed to evaluate locomotor activity at 6, 19, and 24 months old. The experimental parameters assessed are the total distance traveled (meters, m), mean speed (m/s), immobility time (seconds, s), and time spent in the center (seconds, s) (**B**). Nest-building behavior is evaluated in 6-month-old vs. 19-month-old wild-type and NRF2 KO mice by grading quality of nests on a scale from 0 (nestlets untouched) to 5 (near-perfect nests) and by measuring the percentage of shredded nestlets after 12 h of individual housing (**C**). Total body weight (**D**) and skin fold measurements (**E**) are recorded for additional characteristics of aging phenotypes. Kaplan–Meier survival curve indicates the survival rate of wild-type and NRF2 KO mice over a 24-month period (**F**). The data is presented as means ± SD. * indicates statistical significance as determined by one-way ANOVA.

Physical signs of aging were evaluated in the wild-type and NRF2 KO mice at 24 months of age, using 6-month-old adults as controls. Typical phenotypic indicators of aging in mice include fur graying, hair thinning, and a hunched posture. These age-related features were pronounced in the NRF2 KO mice (Figure 6A).

Locomotor and motor activities were assessed using an open field test and nesting behavior assessment. At 19 and 24 months of age, both the wild-type and NRF2 KO mice exhibited significantly reduced travel distances and mean velocities compared with the 6-month-old controls (Figure 6B). The older wild-type and NRF2 KO mice showed an increase in their immobility time relative to the 6-month-old mice, although the increase was only significant in the NRF2 KO mice (Figure 6B). No significant differences were observed in the time spent in the central zone among groups (Figure 6B), excluding anxiety as a potential cause of the reduced morbidity. However, the nest-building activity, another indicator of physiological aging in mice [27], showed a decline in the NRF2 KO mice at 19 months of age (Figure 6C). These findings indicate that while the locomotor activity did not show genotypic-specific decline within 24 months and did not correlate with cardiac aging, NRF2 KO mice are prone, with age, to decline in complex high-order motivated behavior, i.e., nesting.

While no significant difference in body weight was observed between the 6- and 24-month-old wild-type mice, the 24-month-old NRF2 KO mice showed a significant reduction in their body weight compared with the 6-month-old NRF2 KO mice (Figure 6D). The dorsal skin fold thickness was significantly decreased in aged mice of both wild-type and NRF2 KO groups (Figure 6E). The Kaplan–Meier survival analysis revealed a marked reduction in lifespan for the NRF2 KO mice compared to wild-type mice, beginning at 17 months of age (Figure 6F). At 24 months, the wild-type mice exhibited a survival probability of 70%, whereas the NRF2 KO mice showed a reduced survival probability of about 30%. These findings indicate that NRF2 deficiency accelerates aging phenotypes and shortens lifespan compared to wild-type controls.

## 4. Discussion

We examined age-associated changes in the transcription factor NRF2 across four species. Such multi-species analyses help to distinguish fundamental and conserved mechanisms of aging from species-specific biological features. Because each species has inherent limitations, studies using experimental animals complement the largely correlational nature of human aging research. The NRF2-dependent transcriptional network appears to decline with chronological age in the myocardium of humans, monkeys, and rats, as evidenced by reduced NRF2 transcript or protein expression. In humans, NRF2 mRNA levels show a trend toward reduction between 20–29 and 40–49 years of age. Age-associated decreases in the majority of genes within the “Regulation of Translation” GO term support impaired NRF2 protein synthesis with aging in humans. Consistent with reduced NRF2 activity, transcripts of multiple downstream antioxidant and detoxification genes, including SOD1, SOD2, CAT, PRDX1, SRXN1, GCLC, GCLM, UGGT1, and MT1A, show a trend toward or significant decreases with age. In monkey and rat myocardium, NRF2 protein levels decline with age without corresponding reductions in NRF2 mRNA or increases in KEAP1 mRNA, supporting an impairment at the level of NRF2 protein translation. Functionally, the importance of NRF2 in preventing aging-associated decay was demonstrated using NRF2 gene knockout mice. The mice deficient in the NRF2 gene exhibited accelerated cardiac aging, premature heart failure, aging-associated behavioral decline, and a shortened lifespan. Together, these findings support the concept that chronological age is associated with impaired NRF2 signaling and demonstrate the critical role of NRF2 in protecting against cardiac aging.

Analyses of human myocardial gene expression present several challenges that can affect the validity and sensitivity of the data. Studies on human myocardial aging are inherently limited by the use of postmortem tissue samples of donors who died from various causes. A substantial proportion of donors to the GTEx project had a history of cardiovascular disease [18,19,20]. Although the sample collection protocols were carefully standardized to minimize postmortem artifacts, such sources of samples may not accurately represent healthy aging. In addition, uneven group sizes, particularly the small number of samples in the 20–29 and 70–79 age groups, can reduce statistically sensitivity, limiting the ability to detect meaningful differences and producing unstable variance estimates. Moreover, inter-individual variabilities arising from genetic backgrounds, comorbidities, dietary factors, and environmental exposures further increase background noise, reduce statistical power, and complicate the identification of true biological signals. These limitations underscore the importance of larger and more balanced sample sizes, as well as rigorous adjustment for confounding variables, in future population-based transcriptomic studies of human aging.

Aging is a multifactorial biological process that leads to the progressive functional decline of the heart. However, the timeline, severity, and physiological manifestations of aging differ substantially among species. In humans, advancing chronological age is associated with the gradual accumulation of oxidative damage, mitochondrial dysfunction, cellular senescence, impaired proteostasis, and other molecular hallmarks of aging [29,30,31]. These changes develop slowly over decades and contribute to the progressive deterioration of myocardial function and increased susceptibility to cardiovascular disease. Rhesus monkeys exhibit a slower aging trajectory that closely resembles human aging in terms of lifespan and cardiovascular physiology, making them a valuable model for studying cardiac aging. In contrast, mice undergo a much more compressed aging process, with rapid progression of age-associated cardiac changes occurring near the end of their lifespan. Such species-dependent differences in aging kinetics are important considerations when interpreting the molecular and physiological changes associated with chronological aging.

In the present study, wild-type mice at 19 months of age exhibited preserved contractile function accompanied by adaptive cardiac hypertrophy (Figure 5). Overt signs of cardiac functional decline were not apparent even at 24 months of age. In contrast, loss of NRF2 accelerated aging-associated cardiac dysfunction and progression toward heart failure. The NRF2 KO mice displayed reduced contractile performance at 19 months of age. The majority of NRF2 KO mice died by 25–26 months of age, which limited the feasibility of extending the experiments beyond 26 months. Consequently, it was not economic to assess whether the wild-type mice would eventually exhibit a decline in myocardial NRF2 expression at a later age. This limitation may explain, in part, the absence of NRF2 protein reduction in the myocardium of 24-month-old wild-type mice in our study. Nevertheless, we detected a modest reduction in NRF2 mRNA in aged mouse hearts at 24 months, consistent with a recent report demonstrating a clear decline in NRF2 protein by 30 months of age [32]. Compared with humans and nonhuman primates, mice may maintain cardiac health and relatively preserved NRF2 signaling until late in life, followed by a more abrupt deterioration near the terminal phase of aging. Therefore, the apparent absence of a gradual decline in NRF2 protein levels during earlier stages of senescence in mice likely reflects species-specific temporal dynamics of NRF2 regulation rather than a resistance to age-associated NRF2 impairment. These observations further emphasize the value of multi-species analyses for understanding the mechanism of aging in humans. Differences in the lifespans and aging trajectories among species can profoundly influence the timing and manifestation of molecular aging phenotypes. Cross-species comparisons therefore provide critical context for distinguishing conserved mechanisms of cardiac aging from species-dependent adaptations.

A decline in NRF2 expression with aging suggests progressive impairment of endogenous antioxidant defense mechanism in the myocardium. Chronological aging is accompanied by increased oxidative stress, characterized by elevated ROS production and enhanced protein oxidation. Because ROS can promote NRF2 protein translation as well as NRF2 transcriptional activity, increased oxidative stress during aging would be expected to trigger compensatory activation of the NRF2 signaling pathway. The loss of this oxidant-driven NRF2 response provides a compelling explanation for the decline in antioxidant capacity, accumulation of oxidative damage, and increased vulnerability to cardiovascular disease in aging populations.

Oxidative stress is a major contributing factor to the initiation and progression of cardiovascular diseases, including myocardial infarction and heart failure. Compared to younger individuals, older patients typically exhibit larger infarct sizes, a higher incidence of heart failure, and an increased mortality rate [33]. Normally, brief cycles of ischemia and reperfusion can elicit cardioprotective effects, as demonstrated by both clinical and experimental evidence of pre- and post-ischemic conditioning. However, these endogenous cardioprotective mechanisms are diminished in elderly patients or aged experimental animals [34,35,36,37,38,39]. Because ischemic preconditioning activates NRF2 for its activity as a transcription factor [40], age-related reductions in NRF2 gene expression and the inability to synthesize the NRF2 protein may explain the loss of cardioprotection in the elderly.

The role of NRF2 in the oxidative stress response has gained increasing attention as a potential strategy for aging prevention. Age-associated declines in the NRF2 gene and protein expression underscore the importance of restoring NRF2 signaling to enhance myocardial resilience against tissue injury. Among non-pharmacological interventions, moderate exercise has been shown to induce NRF2 activation in the myocardium [41,42,43]. Pharmacological activators of NRF2, including sulforaphane and oleanane triterpenoids such as bardoxolone methyl, have demonstrated cardioprotective effects in experimental models of ischemic reperfusion injury and chronic heart failure [11,44]. However, clinical trials evaluating cardiac protection via NRF2 activation remain lacking. Nevertheless, while appropriate NRF2 activation is broadly beneficial for counteracting oxidative stress, excessive or dysregulated NRF2 activation can be detrimental, leading to reductive stress, impairment of protein quality control, and disruption of nitric oxide signaling [45,46,47,48]. Thus, maintaining an optimal redox balance with adequate NRF2 expression is essential for cardiovascular health.

Our findings are consistent with a number of published studies demonstrating that aging is associated with a decline in the NRF2-mediated antioxidant response in the myocardium of small experimental animals [41,49,50,51]. A reduction in nuclear NRF2 protein has been reported in the hearts of aged C57BL/6/SJ mice, pointing to diminished transcriptional activity of NRF2 [41]. Unlike these earlier studies, our investigation systematically characterized the cardiac contractile function and comprehensive aging phenotypes of senescent NRF2 KO mice, with comparisons to their wild-type counterparts derived from the same heterozygous ancestors. While both the wild-type and NRF2 KO mice showed declines in locomotor activities, such as reduced distance or mean speed of traveling by 19 months old, only the NRF2 KO mice displayed cardiac aging by this age point. This indicates that neuromuscular aging can occur independent of cardiac aging. Although our results suggest a decrease in NRF2 transcriptional activity in the aging myocardium, this pattern is not uniform across tissues. The nuclear NRF2 protein and its downstream target genes, NQO1, GST, and ALDH1, were reported to be elevated in the liver of 24-month-old mice compared to 2-month-old mice [52]. Similarly, vascular smooth muscle cells from aged rats exhibited enhanced NRF2 activity, accompanied by upregulation of multiple antioxidant and detoxification enzymes [53]. Collectively, these findings highlight cell- and tissue-specific differences in the regulation of NRF2 signaling during aging.

## Figures and Tables

**Table 1:** Primer Sequences for PCR.

Gene	Species	Accession #	Forward	Reverse
*Nrf2*	Mouse	010902.5	CATGATGGACTTGGAGTTGC	CCTCCAAAGGATGTCAATCAA
	Rat	031789.3	TTTGTAGATGACCATGAGTCGC	GCCAAACTTGCTCCATGTCC
	Monkey	001257607.1	CCAACACACGGTCCCCAG	ACCTGGGAGTAGTTGGCAGA
*Keap1*	Mouse	016679.4	GATCGGCTGCACTGAACTG	GGTTGAAGAACTCCTCCTGCT
	Rat	* 006242591.5	TGGTTCCTGCAACTTGGTGA	CTATGTGTCCCACAAGGGAGC
	Monkey	* 015122858.3	ACGGGACAAACCGCCTTAAT	GTCCAGGAACGTGTGACCAT
*Nqo1*	Mouse	008706.5	AGCGTTCGGTATTACGATCC	AGTACAATCAGGGCTCTTCTCG
	Rat	017000.3	AGCGCTTGACACTACGATCC	TCTGCGTGGGCCAATACAAT
	Monkey	001260998.1	AGGACCCTGCGAACTTTCAG	CTGGAGTGTGCCCAATGCTA
*Gclm*	Mouse	008129.4	TGGAGCAGCTGTATCAGTGG	CAAAGGCAGTCAAATCTGGTG
	Rat	017305.2	CGCCTGCGGAAAAAGTGTC	CCACTGCATGGGACATGGTA
	Monkey	* 015145343.3	AGCGAGGAGCTTCGTGATTG	GAACAGGCCATGTCAACTGC
*Sqstm1*	Mouse	011018	TTATAGAGCGATACAAGGGGGAG	CGCCGTCTGATTATCTTGATGAG
	Rat	175843.5	CCAGCTGTTTCCTCCGTACC	CCATCCTCATCGCGGTAGTG
	Monkey	001266358.1	AATGACTCGGAGGTCCCTGA	CAGCTCTCAGCGAAACCAAC
*Rpl32*	Monkey	001193573.1	CCTGGAGGAGAAGAGGAAAGAGA	TTGAGGACCTCTGTGTATTTGTCAA
*Actb*	Rat	031144.3	AGTACAACCTTCTTGCAGCTC	TGACCCATACCCACCATCAC
*GAPDH*	Mouse	001289726.2	AGGTCGGTGTGAACGGATTTG	TGTAGACCATGTAGTTGAGGTCA
*ANP*	Mouse	008725.3	GCTTCCAGGCCATATTGGAG	GGGGGCATGACCTCATCTT
*BNP*	Mouse	008726.6	GAGGTCACTCCTATCCTCTGG	GCCATTTCCTCCGACTTTTCT
*β-MHC*	Mouse	001361607.1	ACTGTCAACACTAAGAGGGTCA	TTGGATGATTTGATCTTCCAGGG

All gene accession number starts with NM_, except * starting with XN_.

## Data Availability

All data were generated by our laboratory. Raw data or methods are available upon request.

## Supplementary Materials

The following supporting information can be downloaded at: https://www.mdpi.com/article/10.3390/antiox15060672/s1, Figure S1: NRF2 Protein Sequence Alignment between Human, Monkey, Rat and Mouse Species; Figure S2: Expression of NRF2 Downstream Antioxidant Genes in Human Left Ventricular Tissues; Figure S3: Expression of NRF2 Downstream Detoxification Genes in Human Left Ventricular Tissues; Figure S4: Expression of NRF2 Target Genes Encoding Metal-Binding Proteins in Human Left Ventricular Tissues; Figure S5: Expression of Autophagy Genes in Human Left Ventricular Tissues; Figure S6: Gene Ontology (GO) of the Transcriptomes of Human Left Ventricular Myocardial Tissues; Figure S7: Cardiac Hypertrophy in Aged Wild-Type and NRF2 KO Mice.
