# Supplementary material for: Loss of NRF2 During Aging Contributes to Myocardial Functional Decline
Source: Antioxidants (Basel). 2026 May 27;15(6):672. doi: 10.3390/antiox15060672 (PMC13295359; doi:10.3390/antiox15060672)
Supplement: Supplementary file 1 [file antioxidants-15-00672-s001.zip › antioxidants-4220964-supplementary.pdf]

Fig S1

```
                                                       Neh2
Human     MMDLELPPPGLPSQQDMDLIDILWRQDIDLGVSREVFDFSQRRKEYELEKQKKLEKERQE    60
Monkey    MMELELPPPGLPSQQDMDLIDILWRQDIDLGVSREVFDFSQRRKEYELEKQKKLEKERQE    60
Mouse     MMDLELPPPGLQSQQDMDLIDILWRQDIDLGVSREVFDFSQRQKDYELEKQKKLEKERQE    60
Rat       MMDLELPPPGLQSQQDMDLIDILWRQDIDLGVSREVFDFSQRQKDYELEKQKKLEKERQE    60
          **:******** ******************************:*:***************

Human     QLQKEQEKAFFAQLQLDEETGEFLPIQPAQHIQSETSGSANYSQVAHIPKSDALYFDDCM    120
Monkey    QLQKEQEKAFFAQLQLDEETGEFLPIQPAQHIQSETSGSANYSQVAHIPKSDALYFDDCM    120
Mouse     QLQKEQEKAFFAQFQLDEETGEFLPIQPAQHIQTDTSGSASYSQVAHIPKQDALYFEDCM    120
Rat       QLQKEQEKAFFAQLQLDEETGEFLPIQPAQHIQTDTSGSVSYSQVAHIPKQDALYFEDCM    120
          *************:*******************::****..*********.*****:***

Human     QLLAQTFPFVDDNEVSSATFQSLVPDIPGHIESPVFIATNQAQSPETSVAQVAPVDLDGM    180
Monkey    QLLAQTFPFVDDNEVSSATFQSLVPDIPGHIESPVFIATNQAQSPETSVAQVAPVDLDGM    180
Mouse     QLLAETFPFVDDHE-------SLALDIPSHAESSVFTAPHQAQSLNSSL-EAAMTDLSSI    172
Rat       QLLAETFPFVDDHEVSSPTFQSLALDIPSHVESSVFTTPDQAQSLDSSL-ETAMTDLSSI    179
          ****:*******:*       **. ***.* ** ** : .**** ::*: :.* .**..:

Human     QQDIEQVWEELLSIPELQCLNIENDKLVETTMVPSPEAKLTEVD-NYHFYSSIPSMEKEV    239
Monkey    QQDIEQVWEELLSIPELQCLNIENDKLVETTMVPSPEAKLTEVD-NYHFYSSISSMEKEV    239
Mouse     EQDMEQVWQELFSIPELQCLNTENKQLADTTAVPSPEATLTEMDSNYHFYSSISSLEKEV    232
Rat       QQDMEQVWQELFSIPELQCLNTENKQQAETTTVPSPEATLTEMDSNYHFYSSIPSLEKEV    239
          :**:****:**:********* **.: .:** ******.***:* ******** *:****

Human     GNCSPHFLNAFEDSFSSILSTEDPNQLTVNSLNSDATVNTDFGDEFYSAFIAEPSISNSM    299
Monkey    GNCSPHFLNAFEDSFSSILSTEDPNQLTVNSLNSDATVNTDFGDEFYSAFIAEPSISNSM    299
Mouse     GNCGPHFLHGFEDSFSSILSTDDASQL--TSLDSNPTLNTDFGDEFYSAFIAEPSDGGSM    290
Rat       DSCSPHFLHGFEDSFSSILSTDDASQL--NSLDSNPTLNTDFGDEFYSAFLAEPSGGGSM    297
          ..*.****:.***********:* .**  .**:*: *:***********:**** ..**

Human     PSPATLSHSLSELLNGPIDVSDLSLCKAFNQNHPESTAEFNDSDSGISLNTSPSVASPEH    359
Monkey    PSPATLSHSLSELLNGPIDVSDLSLCKAFNQNHPESTTEFNDSDSGISLNTSPSVASPEH    359
Mouse     PSSAAISQSLSELLDGTIEGCDLSLCKAFNPKHAEGTMEFNDSDSGISLNTSPSRASPEH    350
Rat       PSSAAISQSLSELLGGPIEGCDLSLCKAFNQKHTEGTVEFNDSDSGISLNTSPSRASPEH    357
          ** *::*:******.* *: .********* :* *.* **************** *****

Human     SVESSSYGDTLLGLSDSEVEELDSAPGSVKQNGPKT-PVHSSGDMVQPLSPSQGQSTHVH    418
Monkey    SVESSSYGDTLLGLSDSEVEELDSAPGSVKQNGPKTQPVHSSGDTVQPLSPSQGQSTPVH    419
Mouse     SVESSIYGDPPPGFSDSEMEELDSAPGSVKQNGPKAQPAHSPGDTVQPLSPAQGHSAPMR    410
Rat       SVESSIYGDPPPGFSDSEMEELDSAPGSVKQNGPKAQPTHSSGDTVQPLSPAQGHSAAVH    417
          ***** ***   *:****:****************: *.** ** ******:**:*: ::

Human     DAQCENTPEKELPVSPGHRKTPFTKDKHSSRLEAHLTRDELRAKALHIPFPVEKIINLPV    478
Monkey    DAQCENTPEKELPLSPGHGKTPFTKDKHSSRLEAHLTRDELRAKALHIPFPVEKIINLPV    479
Mouse     ESQCENTTKKEVPVSPGHQKAPFTKDKHSSRLEAHLTRDELRAKALHIPFPVEKIINLPV    470
Rat       ESQCENTTKKEVPVSPGHQKVPFTKDKHSSRLEAHLTRDELRAKALHIPFPVEKIINLPV    477
          ::***** :**:*:**** *.***************************************

Human     VDFNEMMSKEQFNEAQLALIRDIRRRGKNKVAAQNCRKRKLENIVELEQDLDHLKDEKEK    538
Monkey    VDFNEMMSKEQFNEAQLALIRDIRRRGKNKVAAQNCRKRKLENIVELEQDLDHLKDEKEK    539
Mouse     DDFNEMMSKEQFNEAQLALIRDIRRRGKNKVAAQNCRKRKLENIVELEQDLGHLKDEREK    530
Rat       DDFNEMMSKEQFNEAQLALIRDIRRRGKNKVAAQNCRKRKLENIVELEQDLGHLKDEREK    537
           **************************************************.*****:**

Human     LLKEKGENDKSLHLLKKQLSTLYLEVFSMLRDEDGKPYSPSEYSLQQTRDGNVFLVPKSK    598
Monkey    LLKEKGENDKSLHLLKKQLSTLYLEVFSMLRDEDGKPYSPSEYSLQQTRDGNVFLVPKSK    599
Mouse     LLREKGENDRNLHLLKRRLSTLYLEVFSMLRDEDGKPYSPSEYSLQQTRDGNVFLVPKSK    590
Rat       LLREKGENDRNLHLLKRKLSTLYLEVFSMLRDEDGKPYSPSEYSLQQTRDGNVFLVPKSK    597
          **:******:.*****::******************************************

Human     KPDVKKN    605
Monkey    KPDVKKN    606
Mouse     KPDTKKN    597
Rat       KPDTKKN    604
          ***.***
```

Figure S1. **NRF2 Protein Sequence Alignment between Human, Monkey, Rat, and Mouse Species.** NRF2 protein sequences, NP_006155.2 for human, NP_001244536.1 for monkey, NP_001386102.1 for rat, NP_035032.1 for mouse, were retrieved from the NIH National Library of Medicine's National Center for Biotechnology Information database (http://www.ncbi.nlm.nih.gov). These sequences were aligned using Clustal Omega Multiple Sequence Alignment software (http://www.ebi.ac.uk/jdispatcher/msa/clustalo).

Fig S2

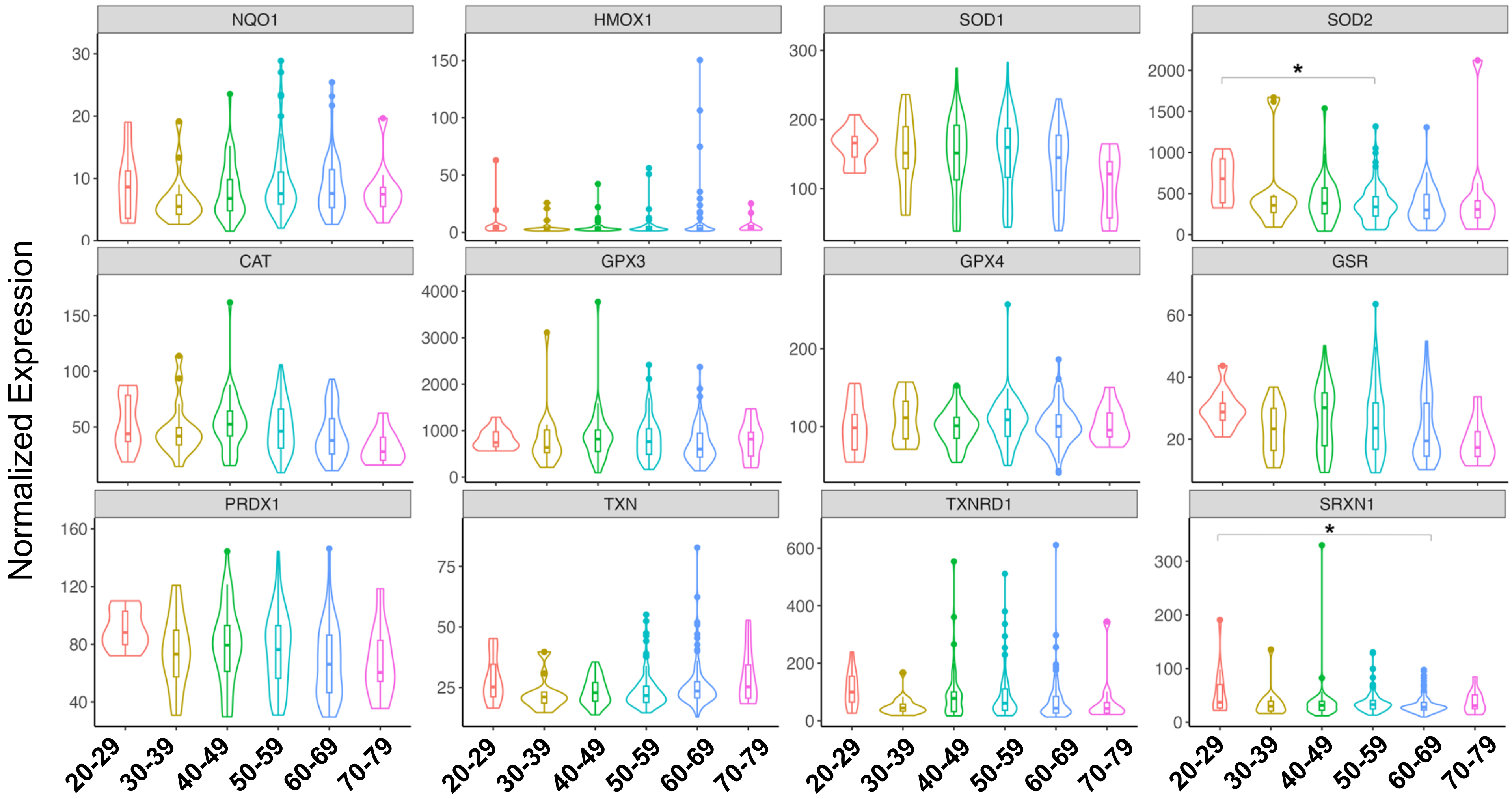
Normalized Expression
NQO1
HMOX1
SOD1
SOD2
CAT
GPX3
GPX4
GSR
PRDX1
TXN
TXNRD1
SRXN1
*
*
20-29
30-39
40-49
50-59
60-69
70-79

Figure S2. **Expression of NRF2 Downstream Antioxidant Genes in Human Left Ventricular Tissues.** Violin plots show distribution of transcripts of antioxidant genes, NQO1, HMOX1, SOD1, SOD2, CAT, GPX3, GPX4, GSR, PRDX1, TXN, TXNRD1, and SRXN1, in each of 6 age groups. The center lines or box limits indicate median or upper and lower quartiles respectively. For pairwise comparison between age groups, the likelihood ratio tests were performed in edgeR. * indicates FDR-adjusted p-value < 0.05.

Fig S3

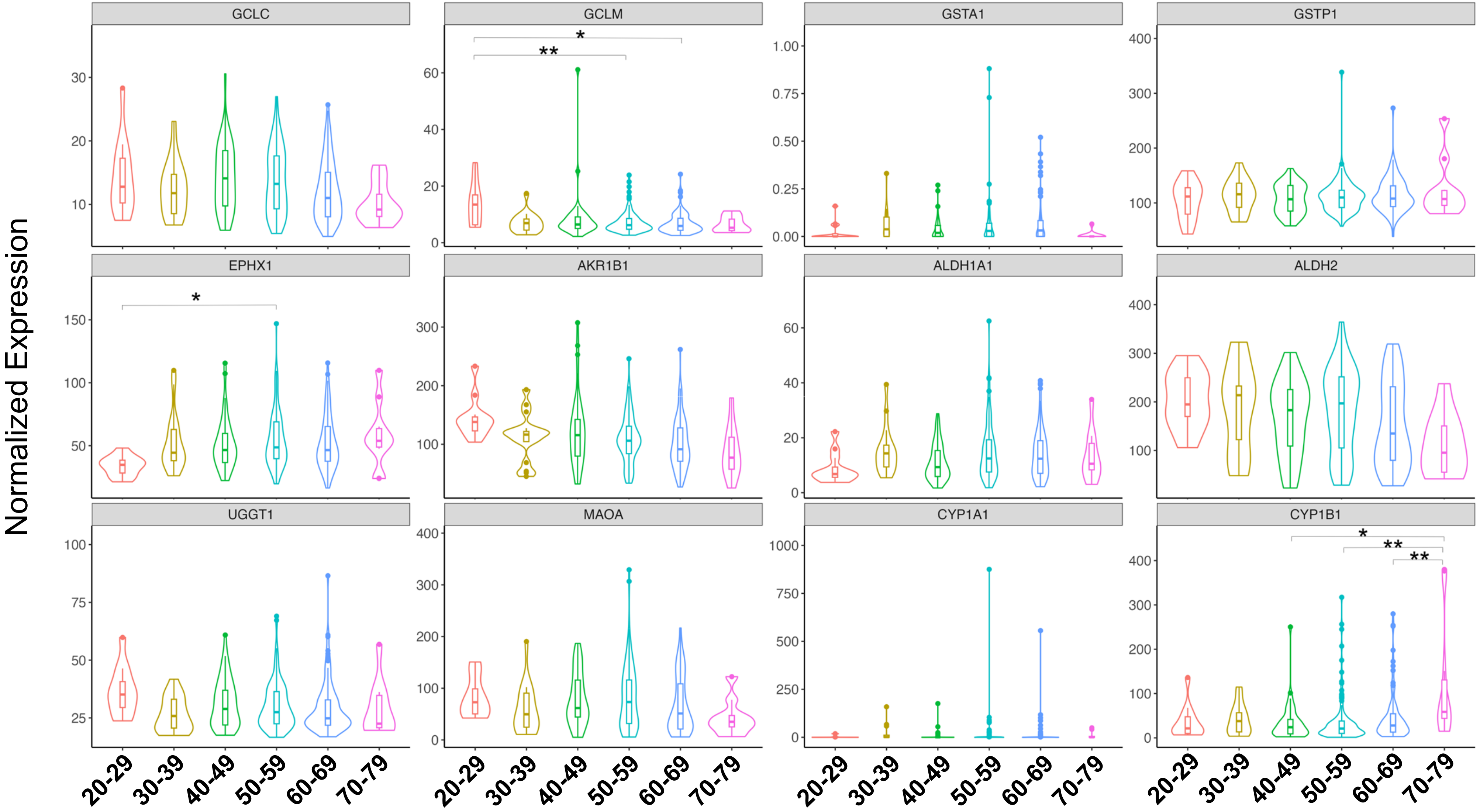
Normalized Expression
GCLC
GCLM
GSTA1
GSTP1
EPHX1
AKR1B1
ALDH1A1
ALDH2
UGGT1
MAOA
CYP1A1
CYP1B1
20-29
30-39
40-49
50-59
60-69
70-79

Figure S3. **Expression of NRF2 Downstream Detoxification Genes in Human Left Ventricular Tissues.** Violin plots display the normalized expression of detoxification genes, GCLC, GCLM, GSTA1, GSTP1, EPHX1, AKR1B1, ALDH1A1, ALDH2, UGGT1, MAOA, CYP1A1, and CYP1B1, across 6 age groups. Median value, upper and lower quartiles, and statistical significance are described in Fig S2. * or ** indicates FDR-adjusted p-value < 0.05 or <0.01, respectively.

Fig S4

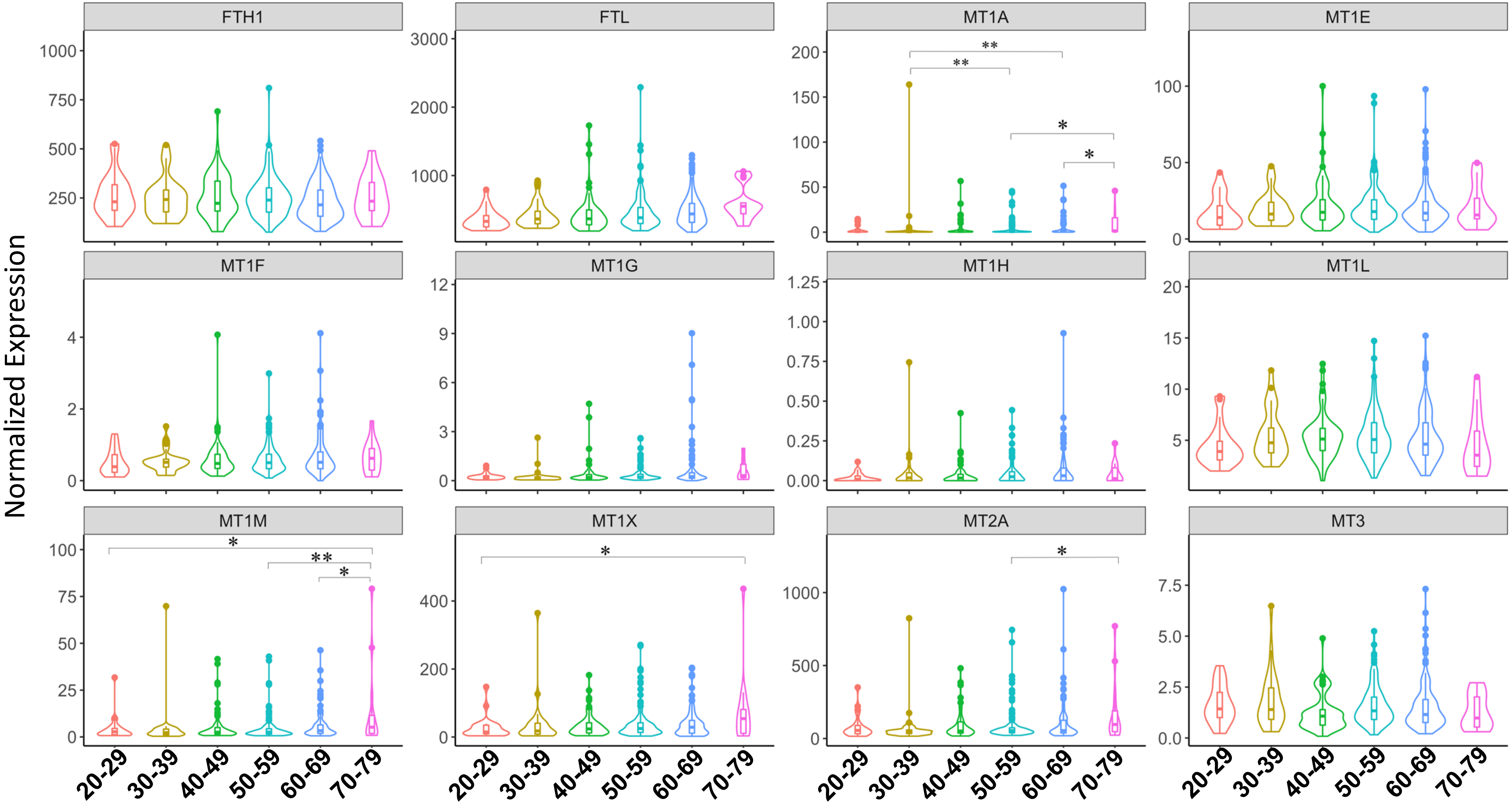

Normalized Expression
FTH1
FTL
MT1A
MT1E
MT1F
MT1G
MT1H
MT1L
MT1M
MT1X
MT2A
MT3
20-29
30-39
40-49
50-59
60-69
70-79

Figure S4. **Expression NRF2 Target Genes Encoding Metal Binding Proteins in Human Left Ventricular Tissues.** Violin plots illustrate the distribution of genes encoding metal binding proteins, FTH1, FTL, MT1A, MT1E, MT1F, MT1G, MT1H, MT1L, MT1M, MT1X, MT2A, and MT3, across 6 age groups. Median value, upper and lower quartiles, and statistical significance are described in Fig S2. * or ** indicates FDR-adjusted p-value < 0.05 or <0.01, respectively.

Fig S5

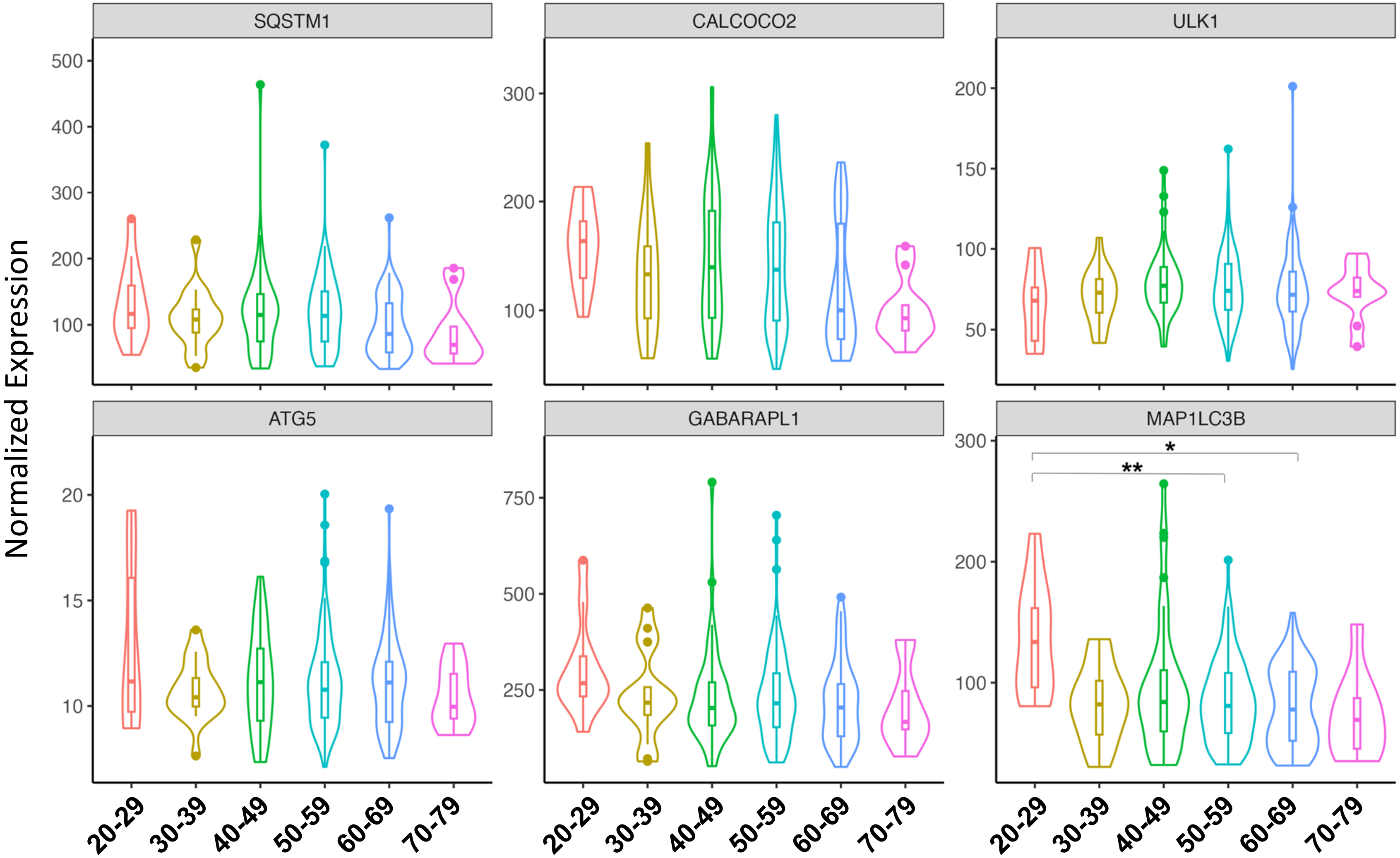
Normalized Expression
SQSTM1
CALCOCO2
ULK1
ATG5
GABARAPL1
MAP1LC3B
*
**
20-29
30-39
40-49
50-59
60-69
70-79

Figure S5. **Expression of Autophagy Genes in Human Left Ventricular Tissues.** Violin plots show transcript levels of autophagy genes, SQSTM1, CALCOCO2, ULK1, ATG5, GABARAPL1, and MAP1LC3B. Statistically significant differences with FDR-adjusted p-value < 0.05 or < 0.01 are indicated by * or **, respectively.

Fig S6

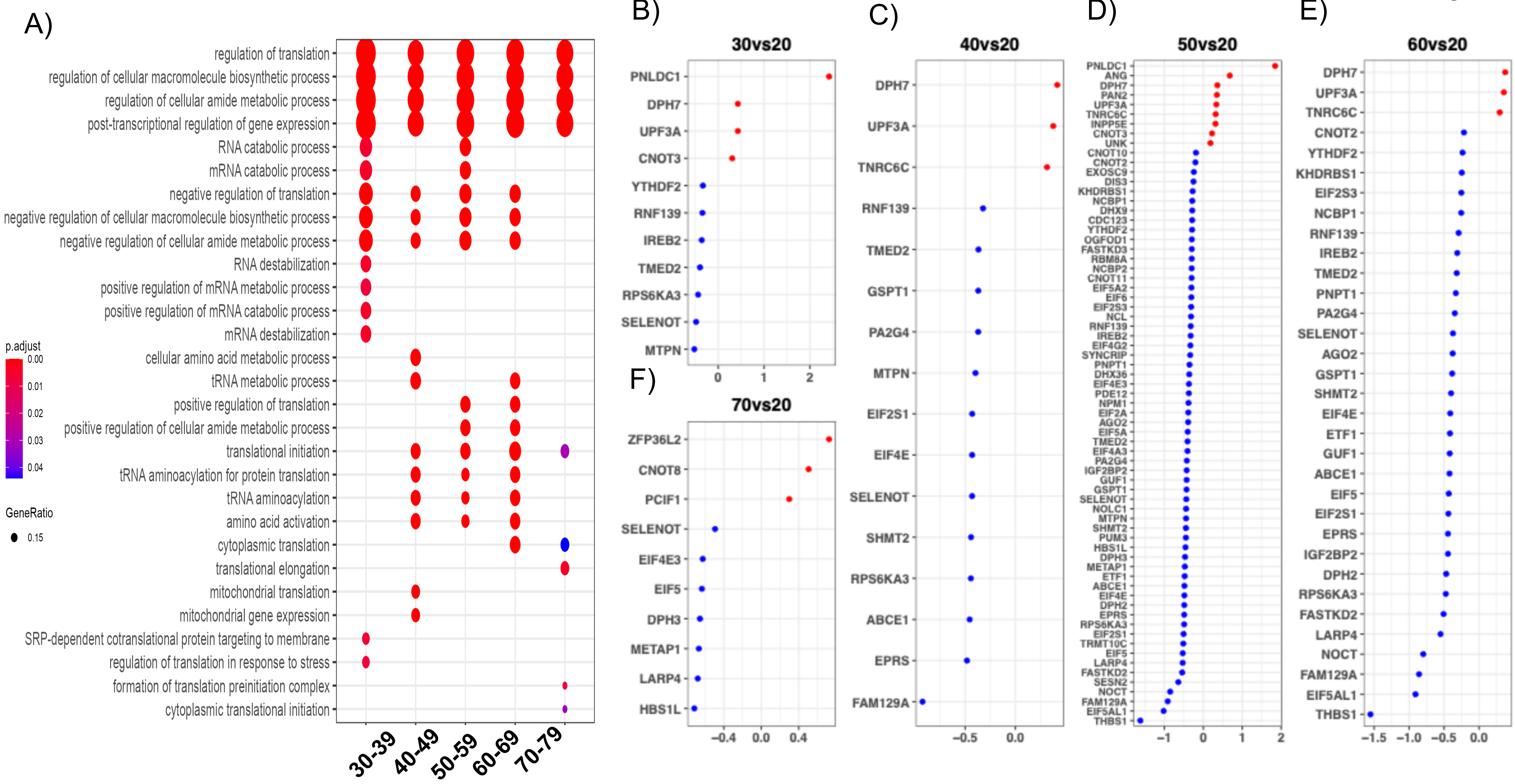
A)
regulation of translation
regulation of cellular macromolecule biosynthetic process
regulation of cellular amide metabolic process
post-transcriptional regulation of gene expression
RNA catabolic process
mRNA catabolic process
negative regulation of translation
negative regulation of cellular macromolecule biosynthetic process
negative regulation of cellular amide metabolic process
RNA destabilization
positive regulation of mRNA metabolic process
positive regulation of mRNA catabolic process
mRNA destabilization
cellular amino acid metabolic process
tRNA metabolic process
positive regulation of translation
positive regulation of cellular amide metabolic process
translational initiation
tRNA aminoacylation for protein translation
tRNA aminoacylation
amino acid activation
cytoplasmic translation
translational elongation
mitochondrial translation
mitochondrial gene expression
SRP-dependent cotranslational protein targeting to membrane
regulation of translation in response to stress
formation of translation preinitiation complex
cytoplasmic translational initiation
30-39
40-49
50-59
60-69
70-79
p.adjust
0.00
0.01
0.02
0.03
0.04
GeneRatio
0.15
B)
30vs20
PNLDC1
DPH7
UPF3A
CNOT3
YTHDF2
RNF139
IREB2
TMED2
RPS6KA3
SELENOT
MTPN
0
1
2
C)
40vs20
DPH7
UPF3A
TNRC6C
RNF139
TMED2
GSPT1
PA2G4
MTPN
EIF2S1
EIF4E
SELENOT
SHMT2
RPS6KA3
ABCE1
EPRS
FAM129A
−0.5
0.0
D)
50vs20
PNLDC1
ANG
DPH7
PAN2
UPF3A
TNRC6C
INPP5E
CNOT3
UNK
CNOT10
CNOT2
EXOSC9
DIS3
KHDRBS1
NCBP1
DHX9
CDC123
YTHDF2
OGFOD1
FASTKD3
RBM8A
NCBP2
CNOT11
EIF5A2
EIF6
EIF2S3
NCL
RNF139
IREB2
EIF4G2
SYNCRIP
PNPT1
DHX36
EIF4E3
PDE12
NPM1
EIF2A
AGO2
EIF5A
TMED2
EIF4A3
PA2G4
IGF2BP2
GUF1
GSPT1
SELENOT
NOLC1
MTPN
SHMT2
PUM3
HBS1L
DPH3
METAP1
ETF1
ABCE1
EIF4E
DPH2
EPRS
RPS6KA3
EIF2S1
TRMT10C
EIF5
LARP4
FASTKD2
SESN2
NOCT
FAM129A
EIF5AL1
THBS1
−1
0
1
2
E)
60vs20
DPH7
UPF3A
TNRC6C
CNOT2
YTHDF2
KHDRBS1
EIF2S3
NCBP1
RNF139
IREB2
TMED2
PNPT1
PA2G4
SELENOT
AGO2
GSPT1
SHMT2
EIF4E
ETF1
GUF1
ABCE1
EIF5
EIF2S1
EPRS
IGF2BP2
DPH2
RPS6KA3
FASTKD2
LARP4
NOCT
FAM129A
EIF5AL1
THBS1
−1.5
−1.0
−0.5
0.0
F)
70vs20
ZFP36L2
CNOT8
PCIF1
SELENOT
EIF4E3
EIF5
DPH3
METAP1
LARP4
HBS1L
−0.4
0.0
0.4

Figure S6. **Gene Ontology (GO) of the Transcriptomes of Human Left Ventricular Myocardial Tissues.** A dot plot displays the GO terms enriched in the transcriptomes of donors in each of the age groups in comparison with that of the 20-29 year old age group **(A)**. The GO enrichment analysis quantified the raw transcript counts. The color gradient represents the adjusted p-values and the size of the dot indicates the GeneRatio which reflects the proportion of input genes associated with each GO term. Individual dot plots show fold change in differentially expressed genes (Y-axis) under the GO term “Regulation of Translation” in comparison with the 20-29 year old age group **(B)**. The X-axis shows the direction and magnitude of change, with the red dots indicating upregulation and blue dots indicating downregulation. The listed genes have an FDR-adjusted p-value of < 0.1.

Fig S7

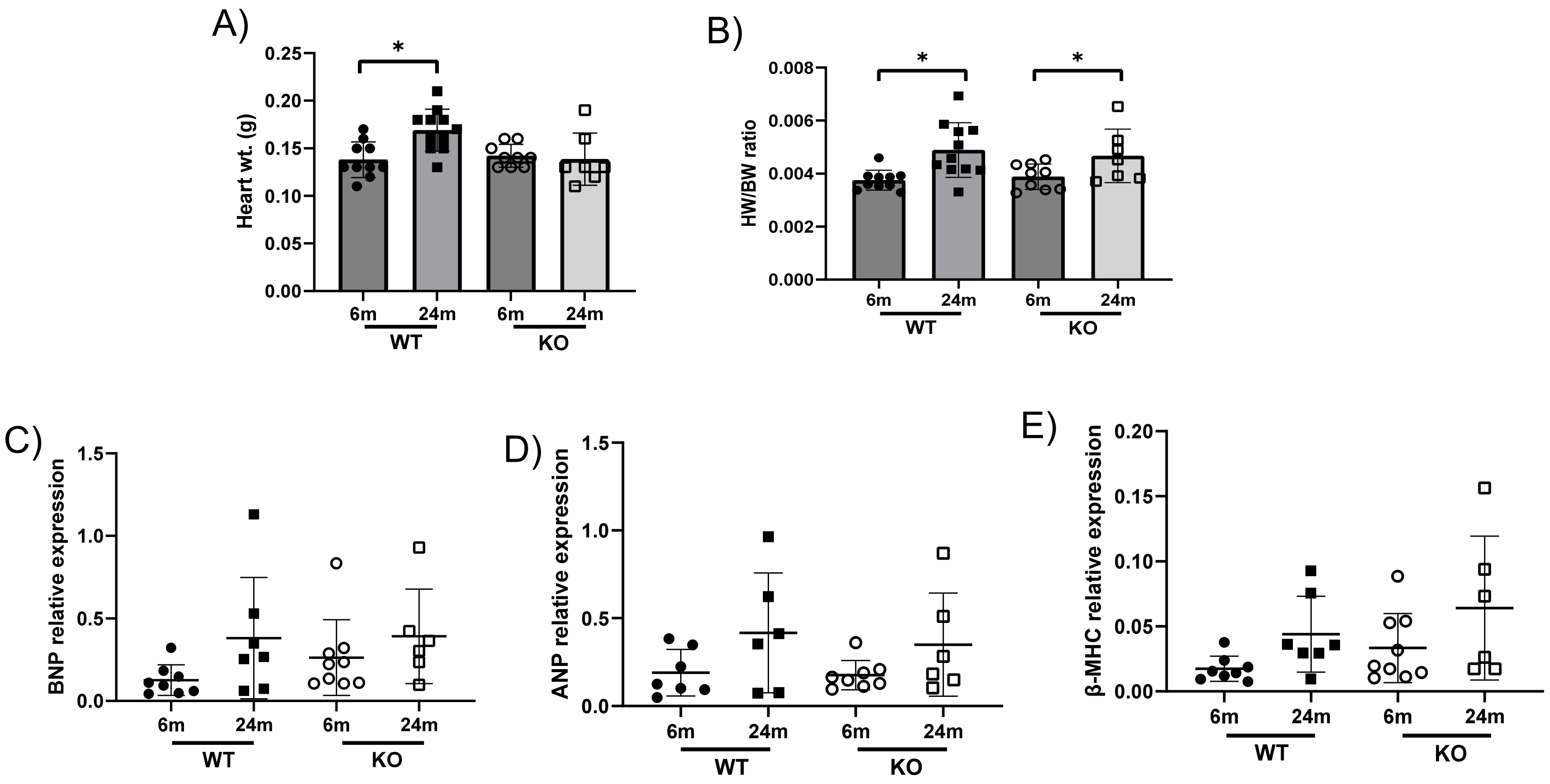
A)
Heart wt. (g)
0.25
0.20
0.15
0.10
0.05
0.00
*
6m
24m
6m
24m
WT
KO
B)
HW/BW ratio
0.008
0.006
0.004
0.002
0.000
*
*
6m
24m
6m
24m
WT
KO
C)
BNP relative expression
1.5
1.0
0.5
0.0
6m
24m
6m
24m
WT
KO
D)
ANP relative expression
1.5
1.0
0.5
0.0
6m
24m
6m
24m
WT
KO
E)
β-MHC relative expression
0.20
0.15
0.10
0.05
0.00
6m
24m
6m
24m
WT
KO

Figure S7. **Cardiac Hypertrophy in Aged Wild Type and NRF2 KO Mice.** Heart weight **(A)** and the ratio of heart to body weight **(B)** were recorded from 6-month-old and 24-month-old mice. ANP, BNP, and β-MHC were measured by RT-qPCR using total RNA extracted from heart ventricular tissues and were normalized to GAPDH **(C-E).** The bar graphs are represented as means ± SD, with * indicating a p-value < 0.05 determined by one-way ANOVA test.
